# Supplementary material for: Exploring cooking fuel choices among Ghanaian women of reproductive age: A socio-economic analysis from a statistical mechanics perspective
Source: PLoS One. 2025 Jan 14;20(1):e0317004. doi: 10.1371/journal.pone.0317004 (PMC11731752; doi:10.1371/journal.pone.0317004)
Supplement: S1 File — (PDF) [file pone.0317004.s002.pdf]

# Supplementary Materials on the Methodology

November 12, 2024

## 0.1 Method

In a population of $N$ women (pregnant and non-pregnant), each woman makes a binary decision, such as either to sleep in an insecticide-treated bed net or not. Each of these binary actions is coded into $\Theta_a$ with

$$\Theta_a = \begin{cases} +1, & \text{if individual } a \text{ sleeps in an intecticide-treated bed net,} \\ -1, & \text{if individual } a \text{ do not sleep in an intecticide-treated bed net .} \end{cases}$$

for a $\in \{1, \cdots, N\}$. We now define the Ising function $H_N$ for any $\Theta \in \Omega_N = \{-1, +1\}^N$ as

$$H_N(\Theta) = \frac{1}{2N} \sum_{a,b=1}^{N} J_{ab} \Theta_a \Theta_b + \sum_{a=1}^{N} h_a \Theta_a. \tag{0.1}$$

The function $H_N$ operating on the configuration $\Theta$ is known as the Hamiltonian or energy function of the model. In statistical mechanics, the function $H_N$ typically arises from the physical properties of a system . In our case, $H_N(\Theta)$ returns the level of satisfaction or utility of the entire population resulting from their choices represented by $\Theta$. Higher values of $H_N(\Theta)$ indicate greater satisfaction or utility within the population. The Hamiltonian $H_N(\Theta)$ consists of two components: the first part models the social incentives of individuals in the population, while the second part models the private incentives of each individual [4]. In this context, $\Theta_a$ represents the choice made by an individual $a$, $J_{ab}$ measures the influence that the individual $a$ has on individual $b$ which represents the strength of social incentive, with $J_{ab} = J_{ba}$ and $h_a$ represents the external field of influence. A positive value of $J_{ab}$ indicates that conformity or imitation is rewarded, whereas a negative value suggests that imitation or conformity is not rewarded [3].

## 0.2 Discrete choice

Our focus now is on modelling the private incentive $h_a$ of an individual. To achieve this, we will initially disregard the interactions by setting $J_{ab} \equiv 0$ for all $a, b \in 1, 2, \cdots, N$, where $N$

represents the total number of individuals in the population. With this simplification, our general utility function can be expressed as follows:

$$H_N(\Theta) = \sum_{a=1}^{N} h_a \Theta_a. \tag{0.2}$$

Each individual will be assigned to a vector of socio-economic attributes $Z_a$ and each component of the vector is a binary variable and takes a value of 1 or 0 [2]. Since each individual has her own distinct socio-economical attribute, we will let $Z_a$ be defined for $a = \{1, \cdots, N\}$ as follows

$$Z_a = \left(Z_a^{(1)}, Z_a^{(2)}, ....Z_a^{(k)}\right) \tag{0.3}$$

where the $Z_a^{(b)}$'s are $\{0,1\}$ - valued for $b = 1, \cdots, k$. For example, take into perspective the situation where the socio-economic characteristics of interest are educational status $Z_a^{(1)}$ and place of residence $Z_a^{(2)}$ with

$$Z_a^{(1)} = \begin{cases} 0, & \text{if individual } a \text{ has some form of education} \\ 1, & \text{if individual } a \text{ has no form of education} \end{cases} \tag{0.4}$$

and

$$Z_a^{(2)} = \begin{cases} 0, & \text{if individual } a \text{ is in rural} \\ 1, & \text{if individual } a \text{ is in urban.} \end{cases} \tag{0.5}$$

If the value of an attribute is zero for an individual, then it means that a particular attribute does not contribute to the private incentive of that individual. Because $h_a$ is what motivates an individual to make a choice or decision, it is reasonable for it to become dependent on the vector of socio-economic attributes $Z_a$ since every rational person considers her status before making a choice or decision. Therefore, it can be written as a function of $Z_a$ as follows: let $\beta_j$ for $j = 0, \cdots, k$ be the component of the vector $\beta = (\beta_0, \beta_1, \cdots, \beta_k)$ and assume $\beta_j$ does not depend on the specific individual $a$. The vector $\beta$ tells us the relative weight or importance that the various socio-economic attributes has or measures the private incentive for each attribute when an individual is making a decision. In our case this leads to

$$h_a = \sum_{j=1}^{k} \beta_j Z_a^{(j)} + \beta_0 \tag{0.6}$$

Here, $\beta_0$ represents the fundamental private incentive that everyone person possesses, irrespective of their unique socio-economic characteristics [3].

## 0.3 The Multipopulation Curie-Weiss Model

This section will concentrate on identifying an appropriate way to express the interaction coefficient $J_{ab}$ and establishing a methodical process for estimating the fundamental parameters of the model using existing data. In our discrete choice model, each person labelled as $a$ is associated with a collection of $k$ socio-economic attributes,

$$Z_a = (Z_a^1, Z_a^2, Z_a^3..., Z_a^k), \text{with } Z_a^{(j)} \in \{0,1\}^k \text{ for } j = 1, 2, \cdots, k. \tag{0.7}$$

As a result, the population of size $N$ can be divided into $2^k$ non-overlapping groups. Each of the groups is identified by one of the elements of $\{0,1\}^k$. For $g = 1, \cdots, 2^k$ and $| I_{N_g} |= N_g$, let $I_{N_g}$ be the collection of people in partition $g$. Therefore $N = N_1 + \ldots + N_{2^k}$. Let $I_N = \bigcup_{g=1}^{2^k} I_{N_g}$ with $I_{N_g} \bigcap I_{N_{g'}} = \varnothing$ for $g \neq g'$ [3].

Individuals who share the same socio-economic attribute are grouped together, and as a result, equation (0.6) implies that all members within a partition or group, denoted as $g$ possess an identical private incentive, denoted as $h_g$. In what follows, we will assume that for any pair of groups $g$ and $g'$, $J_{ab} = J_{gg'}$ for every $a \in I_{N_g}$ and $b \in I_{N_{g'}}$. It follows from this assumption and equation (0.1) that

$$H_N(\Theta) = \frac{1}{2N}\sum_{g=1}^{2^k}\sum_{g'=1}^{2^k}\Big(\sum_{a\in I_{N_g}}\sum_{b\in I_{N_{g'}}} J_{ab}\Theta_a\Theta_b\Big) + \sum_{g=1}^{2^k}\sum_{a\in I_{N_g}} h_a\Theta_a. \tag{0.8}$$

The private incentive component then turns into

$$\sum_{g=1}^{2^k}\sum_{a\in I_{N_g}} h_a\Theta_a = \sum_{g=1}^{2^k}\frac{N_g}{N_g}\sum_{a\in I_{N_g}} h_a\Theta_a = \sum_{g=1}^{2^k} N_g\frac{h_g}{N_g}\sum_{a\in I_{N_g}}\Theta_a = \sum_{g=1}^{2^k} N_g h_g \hat{t_g}. \tag{0.9}$$

We employ the fact that $h_a = h_g$ for every a $\in I_{N_g}$ in the second equality. Note that

$$\hat{t_g} = \frac{1}{N_g}\sum_{a\in I_{N_g}}\Theta_a \tag{0.10}$$

is the average decision for the individuals in group or partition $g$. Again the term in the bracket of the first term in equation (0.8) becomes;

$$\sum_{a\in I_{N_g}}\sum_{b\in I_{N_{g'}}} J_{ab}\Theta_a\Theta_b = \frac{N_g}{N_g}\sum_{a\in I_{N_g}}\Theta_a\frac{N_{g'}}{N_{g'}}\sum_{b\in I_{N_{g'}}} J_{ab}\Theta_b = \hat{J}_{gg'}N_gN_{g'}\hat{t}_g\hat{t}_{g'}.$$

The second equality uses that $J_{ab} = J_{gg'}$ for every $a \in I_{N_g}$, $b \in I_{N_{g'}}$. Now, we can see that by using equation (0.8),

$$H_N(\Theta) = \frac{1}{2N}\sum_{g=1}^{2^k}\sum_{g'=1}^{2^k} J_{gg'}N_g\hat{t_g}N_{g'}\hat{t_{g'}} + \sum_{g=1}^{2^k} h_gN_g\hat{t_g} = N\Big[\sum_{g=1}^{2^k}\sum_{g'=1}^{2^k}\frac{J_{gg'}}{2}\frac{N_g}{N}\hat{t_g}\frac{N_{g'}}{N}\hat{t_{g'}} + \sum_{g=1}^{2^k} h_g\frac{N_g}{N}\hat{t_g}\Big].$$

Let $t_g = \frac{N_g}{N}\hat{t_g}$, $t_{g'} = \frac{N_{g'}}{N}\hat{t_{g'}}$ and $J_{gg'} = \frac{J_{gg'}}{2}$, where $\frac{N_g}{N}$ and $\frac{N_{g'}}{N}$ are the relative sizes of groups $I_{N_g}$ and $I_{N_{g'}}$ respectively. Consequently, $t_g$ and $t_{g'}$ represent the weighted average decision of the members of groups $g$ and $g'$, respectively. Our model has now been changed from individual choices to group choices or decision, hence our general Hamiltonian now becomes;

$$H_N(\Theta) = N\Big[\sum_{g=1}^{2^k}\sum_{g'=1}^{2^k} J_{gg'}t_gt_{g'} + \sum_{g=1}^{2^k} h_gt_g\Big]. \tag{0.11}$$

$H_N$ now signifies the overall satisfaction level within the population. Meanwhile, $J_{gg'}$ assesses the impact that group $g$ has on group $g'$ and serves as a measure of the social motivation shared by groups $g$ and $g'$. A positive value implies that these groups are content with similar choices, either both favouring or disfavouring something. Conversely, a negative value indicates that group imitation is neither encouraged nor rewarding, and $h_g$ denotes the individual motivation within group $g$.

In this study, we will consider using two attributes, educational attainment and place of residence, which implies our number of socio-economic attribute is $k = 2$ and we will have 4 partitions as each attribute assumes two values. We will examine how educational attainment (no education or some form of education) and place of residence (urban or rural) influence the choice of using an insecticide-treated bed net for sleeping or not.

The Hamiltonian (0.11) now becomes

$$H_N(\Theta) = N\Bigg[\sum_{g=1}^{4}\sum_{g'=1}^{4} J_{gg'}t_g t_{g'} + \sum_{g=1}^{4} h_g t_g\Bigg] = N\sum_{g=1}^{4} t_g\Bigg[\sum_{g'=1}^{4} J_{gg'}t_{g'} + h_g\Bigg] = N\Bigg[U_1t_1 + U_2t_2 + U_3t_3 + U_4t_4\Bigg], \tag{0.12}$$

$$\begin{aligned} U_1 &= J_{11}t_1 + J_{12}t_2 + J_{13}t_3 + J_{14}t_4 + h_1, \\ U_2 &= J_{21}t_1 + J_{22}t_2 + J_{23}t_3 + J_{24}t_4 + h_2, \\ U_3 &= J_{31}t_1 + J_{32}t_2 + J_{33}t_3 + J_{34}t_4 + h_3, \\ U_4 &= J_{41}t_1 + J_{42}t_2 + J_{43}t_3 + J_{44}t_4 + h_4. \end{aligned}$$

Individuals are differentiated by their mutual interactions: there are four intra-group interactions $(J_{11}, J_{22}, J_{33}, J_{44})$, tuning how strongly individuals in the same group imitate each other, and twelve inter-group interactions $\big(J_{12}, J_{21}, J_{13}, J_{31}, J_{14}, J_{41}, J_{23}, J_{32}, J_{24}, J_{42}, J_{34}, J_{43}\big)$ ), giving the magnitude of the imitation between individuals from distinct groups.

The case where the model has a parameter value of $k = 1$ was thoroughly analysed in the reference [1]. This study involved establishing the thermodynamic limit of the model and deriving its solution through rigorous methods. Additionally, the paper explored various properties of the model. Notably, it was demonstrated in the same reference that the model exhibits complete factorization, meaning that all information pertaining to its equilibrium state can be fully described by the self-consistent equations presented in [2]:

$$\begin{aligned} \hat{t_1} &= \tanh(J_{11}\hat{t}_1 + J_{12}\hat{t}_2 + J_{13}\hat{t}_3 + J_{14}\hat{t}_4 + h_1) = \tanh(U_1), \\ \hat{t_2} &= \tanh(J_{21}\hat{t}_1 + J_{22}\hat{t}_2 + J_{23}\hat{t}_3 + J_{24}\hat{t}_4 + h_2) = \tanh(U_2), \\ \hat{t_3} &= \tanh(J_{31}\hat{t}_1 + J_{32}\hat{t}_2 + J_{33}\hat{t}_3 + J_{34}\hat{t}_4 + h_3) = \tanh(U_3), \\ \hat{t_4} &= \tanh(J_{41}\hat{t}_1 + J_{42}\hat{t}_2 + J_{43}\hat{t}_3 + J_{44}\hat{t}_4 + h_4) = \tanh(U_4). \end{aligned}$$

In particular, the expectation of $\Theta_a$ for all $a \in g$ is $\hat{t}_g = \tanh(U_g)$, where $\hat{t}_g$ is the expected average decision level for every $g = 1, 2, 3,$ and 4. The general formulation for the $U_g$ in our situation, when $k = 2$, has the following form:

$$U_g = \sum_{g'=1}^{4} J_{gg'} \hat{t}_{g'} + h_g. \tag{0.13}$$

This is a linear regression model and the utility of a group with respect to the parameters $J_{gg'}$ and $\beta_j$'s. Recall that

$$h_g = \sum_{j=1}^{2} \beta_j Z_g^{(j)} + \beta_0.$$

This is the basic quantity needed to estimate the interacting model starting from real data. Here $J_{gg'}$, $\beta_j$, and $\beta_0$ are the parameters to be estimated.

The version of the model presented in equation (0.2) that does not involve interactions is obtained when $J_{gg'} = 0$ for every $g, g'$. The non-interacting Hamiltonian could be expressed similarly by

$$H_N(\Theta) = N \sum_{g=1}^{2^k} h_g t_g. \tag{0.14}$$

This reduces to the following in our case where $k = 2$.

$$H_N(\Theta) = N[h_1 t_1 + h_2 t_2 + h_3 t_3 + h_4 t_4] = N[U_1 t_1 + U_2 t_2 + U_3 t_3 + U_4 t_4]$$

where $U_1 = h_1, U_2 = h_2, U_3 = h_3, U_4 = h_4$. Hence, we can say that $U_g = h_g$ for $g = 1, \cdots, 4$.

The non-interacting model also factorises completely to produce equilibrium weighted average choices:

$$\hat{t}_1 = \tanh U_1$$
$$\hat{t}_2 = \tanh U_2$$
$$\hat{t}_3 = \tanh U_3$$
$$\hat{t}_4 = \tanh U_4.$$

Here $U_g = h_g$. This will be the main tool that we will use to estimate the non- interacting model parameters starting from real statistical data.

## 0.4 Estimation

The least squares method is used to estimate the model parameters. As a result, we must identify the parameter settings that minimize

$$\sum_g \left[\hat{t}_g - \tanh(U_g)\right]^2 \tag{0.15}$$

where $\hat{t_g}$ is the average choice of group $g$. Because $\tanh(U_g)$ is non-linear, the computation will take an extremely long time , see [3]. In the interaction scenario, the independent variables are correlated. As a result, the least squares method is rendered ineffective. In that case, the partial least squares estimation method will be utilized.

Table 1: **Socio-economic attributes of women.**

| Attribute | | | | |
|---|---|---|---|---|
| | Educational attainment ($Z_a^{(1)}$) | | Residence($Z_a^{(2)}$) | |
| Cases | Some form of education | No education | Urban | Rural |
| 1 | 0 | 1 | 1 | 0 |
| 2 | 1 | 0 | 0 | 1 |
| 3 | 0 | 1 | 0 | 1 |
| 4 | 1 | 0 | 1 | 0 |

Table 2: **Population classification based on attributes**

| Attributes | Some form of education | No education |
|---|---|---|
| Rural | 1206 | 628 |
| Urban | 972 | 198 |

Table 3: **Sleeps in an insecticide-treated bed net**

| Attributes | Some form of education | No education |
|---|---|---|
| Rural | 1103 | 549 |
| Urban | 776 | 158 |

Table 4: **Do not sleep in an insecticide-treated bed net**

| Attributes | Some Form of education | No education |
|---|---|---|
| Rural | 103 | 79 |
| Urban | 196 | 40 |

From Tables 3 and 4 we will derive our weighted averages for the various groups. We recall that the weighted average is $t_g = \frac{N_g}{N}\hat{t_g}$ and if $\hat{t_g} = \dfrac{1}{N_g}\sum_{a\in I_{N_g}} \Theta_a$ is substituted into it we get that

$$t_g = \frac{1}{N} \sum_{a \in I_{N_g}} \Theta_a = \frac{1}{N} \left( N_g^{Treated\ bed\ users} - N_g^{Non-treated\ bed\ users} \right), \tag{0.16}$$

where $N_g^{Treated\ bed\ users}$ is the number of people in group $g$ who sleeps in an insecticide-treated bed net and $N_g^{Non-treated\ bed\ users}$ is the number of people in group $g$ who do not sleep in an insecticide-treated bed net, for $g$ running from 1 to 4.

In particular, $g = 1:$ women in the rural area with some form of education

$$t_1 = \frac{1}{3004}(1103 - 103) = \frac{250}{751}$$

$g = 2:$ women in the rural area with no education

$$t_2 = \frac{1}{3004}(549 - 79) = \frac{235}{1502}$$

$g = 3:$ women in the urban area with some from of education

$$t_3 = \frac{1}{3004}(776 - 196) = \frac{145}{751}$$

$g = 4:$ women in the urban area with no education

$$t_4 = \frac{1}{3004}(158 - 40) = \frac{59}{1502}$$

# References


[1] Gallo, I., & Contucci, P. (2007). Bipartite mean field spin systems. Existence and solution. arXiv preprint arXiv:0710.0800.

[2] Gallo, I., Barra, A., & Contucci, P. (2009). Parameter evaluation of a simple mean-field model of social interaction. Mathematical Models and Methods in Applied Sciences, 19(supp01), 1427-1439.

[3] Opoku, A. A., Osabutey, G., & Kwofie, C. (2019). Parameter evaluation for a statistical mechanical model for binary choice with social interaction. Journal of Probability and Statistics, 2019.

[4] Selinger, J. V. (2016). Introduction to the theory of soft matter: from ideal gases to liquid crystals (pp. 131-182). Berlin, Germany: Springer International Publishing.
