# Supplementary material for: Exploring cooking fuel choices among Ghanaian women of reproductive age: A socio-economic analysis from a statistical mechanics perspective
Source: PLoS One. 2025 Jan 14;20(1):e0317004. doi: 10.1371/journal.pone.0317004 (PMC11731752; doi:10.1371/journal.pone.0317004)
Supplement: Attachment — Submitted filename: PONE-D-24-15863_reviewer.pdf [file pone.0317004.s003.pdf]

PLOS ONE

Exploring Cooking Fuel Choices among Ghanaian Women of Reproductive Age: A Socio-Economic Analysis from a Statistical Mechanics Perspective

--Manuscript Draft--

**Manuscript Number:**	PONE-D-24-15863
**Article Type:**	Research Article
**Full Title:**	Exploring Cooking Fuel Choices among Ghanaian Women of Reproductive Age: A Socio-Economic Analysis from a Statistical Mechanics Perspective
**Short Title:**	Exploring Cooking Fuel Choices: A Statistical Mechanics Perspective
**Corresponding Author:**	Richard Kwame Ansah, PhD<br>University of Energy and Natural Resources<br>Sunyani, GHANA
**Keywords:**	Partial least squares, Liquefied Petroleum Gas (LPG), Social Interaction, Women of reproductive age, Ghana
**Abstract:**	Access to clean and efficient cooking fuel is crucial for promoting good health, safeguarding the environment, and driving economic growth. Despite efforts to promote the adoption of cleaner alternatives, traditional solid fuels such as charcoal and firewood remain prevalent in Ghana. In this study, we utilized a statistical mechanical model as a framework to explore the statistical relationship between socio-economic factors, such as educational attainment, wealth status, place of residence, and cooking fuel choices. We analyzed data from the Ghana Malaria Indicator Survey (GMIS) conducted in 2019, involving a total of 2,942 women of reproductive age. Among these, 2.92% of women in rural areas and 10.37% of women in urban areas, both with some form of education, as well as 2.58% of women in rural areas and 10.54% of women in urban areas who were not poor, opted to use LPG for cooking. Conversely, 37.15% of women in rural areas and 22.09% of women in urban areas, both with some form of education, and 3.37% of women in rural areas and 10.47% of women in urban areas who were not poor, opted to use non-LPG fuels for cooking. Our findings showed that in the absence of social interaction, a woman's wealth status has a relationship to her choice of fuel for cooking. Additionally, women of reproductive age in rural areas with some education demonstrated a significant private incentive (40.12%) to use LPG, implying a positive correlation between education and the use of LPG for cooking. However, when social interactions are considered, factors such as education, wealth status, and place of residence have significant relationships with a woman's decision about fuel choice. The interaction strength among women of reproductive age in urban areas with some education shows a negative estimate (-4.06%), suggesting that there is no significant imitative effect. The study further suggests that urban women of reproductive age who are poor exert a greater influence on their urban counterparts who are not poor when social interaction is incorporated. Women of reproductive age in rural areas with some form of education exert a greater influence on women of reproductive age in rural areas with no form of education. We recommend that the government of Ghana and its stakeholders focus on leveraging the influence of urban poor women and educated rural women through community-led programs and educational campaigns. Financial support mechanisms like microfinance and subsidies, alongside reliable LPG infrastructure, can make access easier for these target groups. Tailored communication strategies, peer-to-peer learning, and collaboration with local institutions are crucial for spreading awareness and encouraging the adoption of LPG.
**Order of Authors:**	Richard Kwame Ansah, PhD
	Richard Kena Boadi, PhD
	William Obeng-Denteh, PhD
	Killian Asampana Asosega
	Kassim Tawiah
**Additional Information:**	

Powered by Editorial Manager® and ProduXion Manager® from Aries Systems Corporation

Question	Response
**Financial Disclosure**<br><br>Enter a financial disclosure statement that describes the sources of funding for the work included in this submission. Review the submission guidelines for detailed requirements. View published research articles from *PLOS ONE* for specific examples.<br><br>This statement is required for submission and **will appear in the published article** if the submission is accepted. Please make sure it is accurate.<br><br>**Funded studies**<br>Enter a statement with the following details:<br>• Initials of the authors who received each award<br>• Grant numbers awarded to each author<br>• The full name of each funder<br>• URL of each funder website<br>• Did the sponsors or funders play any role in the study design, data collection and analysis, decision to publish, or preparation of the manuscript?<br><br>Did you receive funding for this work?	The author(s) received no specific funding for this work.
**Competing Interests**<br><br>Use the instructions below to enter a competing interest statement for this submission. On behalf of all authors, disclose any competing interests that could be perceived to bias this work—acknowledging all financial support and any other relevant financial or non-financial competing interests.<br><br>This statement is required for submission and **will appear in the published article** if the submission is accepted. Please make sure it is accurate and that any funding sources listed in your Funding Information later in the submission form are also	The author declares that he has no competing interests.

declared in your Financial Disclosure statement.<br><br>View published research articles from *PLOS ONE* for specific examples.<br><br>**NO authors have competing interests**<br><br>Enter: *The authors have declared that no competing interests exist.*<br><br>**Authors with competing interests**<br><br>Enter competing interest details beginning with this statement:<br><br>*I have read the journal's policy and the authors of this manuscript have the following competing interests: [insert competing interests here]*<br><br>* typeset	
**Ethics Statement**<br><br>Enter an ethics statement for this submission. This statement is required if the study involved:<br><br>• Human participants<br>• Human specimens or tissue<br>• Vertebrate animals or cephalopods<br>• Vertebrate embryos or tissues<br>• Field research<br><br>Write "N/A" if the submission does not require an ethics statement.<br><br>General guidance is provided below. Consult the submission guidelines for detailed instructions. **Make sure that all information entered here is included in the Methods section of the manuscript.**	The ethical protocols for the 2019 Ghana Maternal Health Survey (GMIS) received approval from the Ghana Health Service Ethical Review Committee and the ICF International Institutional Review Board. Participation in this survey was entirely voluntary, with participants being fully informed about the potential risks and benefits involved. Only those who consented to participate by signing an informed consent form were included in the study. There was no specific ethics ID or approval number assigned. Participants were required to give their informed consent before they could take part in the study.

Powered by Editorial Manager® and ProduXion Manager® from Aries Systems Corporation

**Format for specific study types**<br><br>**Human Subject Research (involving human participants and/or tissue)**<br>• Give the name of the institutional review board or ethics committee that approved the study<br>• Include the approval number and/or a statement indicating approval of this research<br>• Indicate the form of consent obtained (written/oral) or the reason that consent was not obtained (e.g. the data were analyzed anonymously)<br><br>**Animal Research (involving vertebrate animals, embryos or tissues)**<br>• Provide the name of the Institutional Animal Care and Use Committee (IACUC) or other relevant ethics board that reviewed the study protocol, and indicate whether they approved this research or granted a formal waiver of ethical approval<br>• Include an approval number if one was obtained<br>• If the study involved *non-human primates*, add *additional details* about animal welfare and steps taken to ameliorate suffering<br>• If anesthesia, euthanasia, or any kind of animal sacrifice is part of the study, include briefly which substances and/or methods were applied<br><br>**Field Research**<br><br>Include the following details if this study involves the collection of plant, animal, or other materials from a natural setting:<br>• Field permit number<br>• Name of the institution or relevant body that granted permission	
**Data Availability**<br><br>Authors are required to make all data underlying the findings described fully available, without restriction, and from the time of publication. PLOS allows rare exceptions to address legal and ethical concerns. See the PLOS Data Policy and FAQ for detailed information.	Yes - all data are fully available without restriction

A Data Availability Statement describing where the data can be found is required at submission. Your answers to this question constitute the Data Availability Statement and **will be published in the article**, if accepted.<br><br>**Important:** Stating 'data available on request from the author' is not sufficient. If your data are only available upon request, select 'No' for the first question and explain your exceptional situation in the text box.<br><br>Do the authors confirm that all data underlying the findings described in their manuscript are fully available without restriction?	
**Describe where the data may be found in full sentences. If you are copying our sample text, replace any instances of XXX with the appropriate details.**<br><br>• If the data are **held or will be held in a public repository**, include URLs, accession numbers or DOIs. If this information will only be available after acceptance, indicate this by ticking the box below. For example: *All XXX files are available from the XXX database (accession number(s) XXX, XXX.*).<br>• If the data are all contained **within the manuscript and/or Supporting Information files**, enter the following: *All relevant data are within the manuscript and its Supporting Information files.*<br>• If neither of these applies but you are able to provide **details of access elsewhere**, with or without limitations, please do so. For example:<br><br>*Data cannot be shared publicly because of [XXX]. Data are available from the XXX Institutional Data Access / Ethics Committee (contact via XXX) for researchers who meet the criteria for access to confidential data.*<br><br>*The data underlying the results presented in the study are available from (include the name of the third party*	The dataset used in this study is included in the supplementary file.

and contact information or URL).<br>• This text is appropriate if the data are owned by a third party and authors do not have permission to share the data.<br><br>* typeset	
Additional data availability information:	

Manuscript

Click here to access/download;Manuscript;plos_latex_template.pdf

Exploring Cooking Fuel Choices among Ghanaian Women of Reproductive Age: A Socio-Economic Analysis from a Statistical Mechanics Perspective

Richard Kwame Ansah[1,2,◕,*], Richard Kena Boadi[2◕], William Obeng-Denteh[2◕], Killian Asampana Asosega[2,3◕] Kassim Tawiah[1,3◕]

[1] Department of Mathematics and Statistics, University of Energy and Natural Resources, Sunyani, Ghana
[2] Department of Mathematics, Kwame Nkrumah University of Science and Technology, Kumasi, Ghana
[3]Department of Statistics and Actuarial Science, Kwame Nkrumah University of Science and Technology, Kumasi,Ghana

◕These authors contributed equally to this work.

* Corresponding author E-mail: richard.ansah@uenr.edu.gh

Abstract

Access to clean and efficient cooking fuel is crucial for promoting good health, safeguarding the environment, and driving economic growth. Despite efforts to promote the adoption of cleaner alternatives, traditional solid fuels such as charcoal and firewood remain prevalent in Ghana. In this study, we utilized a statistical mechanical model as a framework to explore the statistical relationship between socio-economic factors, such as educational attainment, wealth status, place of residence, and cooking fuel choices. We analyzed data from the Ghana Malaria Indicator Survey (GMIS) conducted in 2019, involving a total of 2,942 women of reproductive age. Among these, 2.92% of women in rural areas and 10.37% of women in urban areas, both with some form of education, as well as 2.58% of women in rural areas and 10.54% of women in urban areas who were not poor, opted to use LPG for cooking. Conversely, 37.15% of women in rural areas and 22.09% of women in urban areas, both with some form of education, and 3.37% of women in rural areas and 10.47% of women in urban areas who were not poor, opted to use non-LPG fuels for cooking. Our findings showed that in the absence of social interaction, a woman's wealth status has a relationship to her choice of fuel for cooking. Additionally, women of reproductive age in rural areas with some education demonstrated a significant private incentive (40.12%) to use LPG, implying a positive correlation between education and the use of LPG for cooking. However, when social interactions are considered, factors such as education, wealth status, and place of residence have significant relationships with a woman's decision about fuel choice. The interaction strength among women of reproductive age in urban areas with some education shows a negative estimate (-4.06%), suggesting that there is no significant imitative effect. The study further suggests that urban women of reproductive age who are poor exert a greater influence on their urban counterparts who are not poor when social interaction is incorporated. Women of reproductive age in rural areas with some form of education exert a greater influence on women of reproductive age in rural areas with no form of education. We recommend that the government of Ghana and its

stakeholders focus on leveraging the influence of urban poor women and educated rural women through community-led programs and educational campaigns. Financial support mechanisms like microfinance and subsidies, alongside reliable LPG infrastructure, can make access easier for these target groups. Tailored communication strategies, peer-to-peer learning, and collaboration with local institutions are crucial for spreading awareness and encouraging the adoption of LPG.

Keywords: Partial least squares, Liquefied Petroleum Gas (LPG), Social Interaction, Women of reproductive age, Ghana.

Introduction

The fuel chosen for daily household activities like food preparation and heating is a routine issue faced by women. They often spend a significant amount of time collecting and cooking with traditional fuels, such as biomass (firewood, charcoal, and other crop residues) [1], [2].

Globally, many low- and middle-income countries (LMICs) rely exclusively on solid fuels (firewood and biomass) for domestic needs. It is estimated that around 650 million people in sub-Saharan Africa (SSA) will depend on these fuels in unhealthy ways until 2040 [3]. The World Health Organization (WHO) survey of 18 African countries revealed that over 90% of households use solid fuels for lighting, heating, and cooking [4]. In Ghana, approximately 80% of households rely on traditional fuels [5].

The persistent use of traditional fuels has detrimental health and environmental effects, especially in developing countries like Ghana [6]. This fuel consumption contributes to land degradation, especially in densely populated areas. Charcoal production which is prevalent in the Savannah zone of northern Ghana, exacerbates deforestation [7]. From 2000 to 2015, Ghana experienced an average annual deforestation rate of 0.3% [4]. Burning these fuels also increases greenhouse gas (GHG) emissions [8] and black carbon [9]. A significant portion of the Ghanaian population has been exposed to harmful particulate matter levels, surpassing WHO recommended limits [4].

Using solid fuels daily contributes to both outdoor and indoor air pollution in LMICs [4]. The WHO attributes the deaths of seven million people globally to exposure to these pollutants [4]. Pollution from unclean cooking fuel was linked to nearly four million premature deaths in 2016 which is more than the combined deaths from tuberculosis, malaria, and HIV [10], [4].

Prolonged exposure to air pollution from traditional cooking fuels poses severe health risks, especially for women and sometimes their children [10]. Such exposure during pregnancy is associated with increased risks of low birth weight, stillbirths, neonatal mortality, and pregnancy-induced hypertensive disorders [11], [12], [13]. Regular inhalation of particulates can infiltrate maternal bloodstreams, leading to cell damage and inflammation, among other issues [14].

The myriad of adverse effects from the constant use of traditional cooking fuels underscores the urgency of transitioning to cleaner and more sustainable energy sources. The Ghanaian government's efforts to promote cleaner cooking fuels led to the introduction of the Rural Liquefied Petroleum Gas (LPG) Promotion and the Cylinder Recirculation Model programs [15]. Financial incentives for LPG transporters in rural areas and government subsidies were launched to make LPG more accessible [16].

However, despite these efforts, under 25% of Ghanaian households use LPG or electricity [17], [18]. This limited uptake could hinder Ghana's pursuit of the Sustainable Development Goals (SDGs), particularly, ensuring universal access to clean energy (SDG 7), combating climate change (SDG 13), fostering sustainable communities (SDG 11), and promoting health and well-being (SDG 3) by 2030.

In India, Gould and Urpelainen [19] noted the importance of clean cooking fuels in reducing indoor air pollution and improving public health. They found that LPG adoption correlates strongly with education, but not necessarily with positive attitudes toward clean fuels [19]. Despite the clear health benefits of clean cooking fuels, gender inequality and education gaps hinder full appreciation and adoption [20].

This paper focuses on understanding the intricate interactions among women within a reference group and how these relationships influence their collective actions [21], [22], [23]. Self-organization, a facet of group behaviour, is evident in various contexts such as biology, ecology, and socio-economic systems [24], [25], [26]. In these situations, even minor alterations to the socio-economic landscape can lead to significant changes in the behaviour of a large group. For example, the introduction of a small immigrant group could result in changes in language accents, whereas strategic interventions by authorities might substantially decrease criminal activities [27], [28], [26].

The idea that large-scale behaviours emerge from changes in the interactions among their components has roots in statistical mechanics. Spin models, especially those explaining ferromagnetism, have demonstrated the phenomenon of phase transitions, as highlighted in [29] and [30]. The mean-field Ising model, introduced in [29], stands as a prime example of these models. Its application extends across diverse fields such as social studies, finance, chemistry, and ecology [31], [32], [33], and [34]. The variant of the multi-population mean-field Ising model, has gained popularity, particularly in studying magnetism in anisotropic substances, as described in [35]. Additionally, scholars in mathematical physics, including Contucci, Gallo, and Barra, have utilized this model in their research, as cited in [36] and [37]. The significance of integrating statistical mechanical models into social science research is underscored by insights from [21], [22], and [23]. This integration offers a valuable avenue for scholars to further explore the profound effects of social interactions on societal dynamics, as suggested in [26].

The Curie-Weiss model for multiple populations provides an analytical framework to study binary decisions. This includes choices such as whether to pursue further education or enter the workforce. These decisions are influenced by the individual's socio-economic environment, leading to the observation that individuals with similar socio-economic backgrounds tend to exhibit comparable decision-making patterns. In contrast, those from different backgrounds are likely to make different choices. This premise has been supported by prior research, as detailed in studies by [37]. Further exploration has been conducted into a multigroup version of this model, as seen in works by [36] and [37]. These studies extended the use of the modified Curie-Weiss approach to multiple demographic groups, ensuring a constant proportion within each group, regardless of overall population size. For example, [37] applied this framework to analyse factors such as suicidal tendencies and marriage trends in Italy, with a specific focus on living location as the socio-economic factor. Additionally, research by [26] has examined the impact of variables such as gender and domicile on educational outcomes across five emerging nations in Africa.

For a deeper exploration of the potential applications of statistical mechanical models in social sciences and energy domains, we suggest consulting the following

references: [21], [22], [23], and [38]. While prior findings have studied factors affecting the choice of cooking fuels in Ghana, few have focused on women of reproductive age [39], [40]. Our unique contribution is to identify a statistical relationship among educational attainment, wealth status, and place of residence in relation to cooking fuel choices. To achieve this, we employ the multipopulation Curie-Weiss model to study the cooking fuel preferences of Ghanaian women of reproductive age.

Methods

Study Design and Data

The study introduces an analytical cross-sectional analysis focusing on females throughout Ghana's original ten regions. This research utilized the 2019 Ghana Malaria Indicator Survey (GMIS) data, which is grounded in the 2010 Population and Housing Census (PHC) conducted by the Ghana Statistical Service (GSS). It's noteworthy that Ghana experienced a regional restructuring in 2019, expanding from 10 to 16 regions, adding 260 more districts and municipalities. However, the 2019 GMIS only covered the original ten regions as delineated in the 2010 PHC due to the new regions' recent establishment. The purpose of the GMIS is to gather detailed information regarding malaria and its effects, providing essential insights for both the national and regional levels, specifically for the ten regions established in the 2010 PHC: Western, Central, Greater Accra, Volta, Eastern, Ashanti, Brong Ahafo, Northern, Upper East, and Upper West. The survey utilized the census enumeration areas (EAs) from the 2010 PHC, as provided by the GSS [41].

The 2019 GMIS study utilized a two-stage sampling process, segmented into 20 distinct groups based on the urban-rural classification in each region. Initially, 200 Enumeration Areas (EAs) were selected, split into 97 urban and 103 rural EAs, following a method of independent choice and size consideration in each group, as elaborated in [41]. In the subsequent stage, 30 households were chosen from each group, cumulatively amounting to 6,000 households. The selection framework within each group was arranged for implicit stratification and proportionate distribution at more granular administrative levels. For an in-depth understanding of the 2019 GMIS sampling, inclusive of data gathering techniques, instruments, and quality assurance, the full report is available in [41]. This study's analysis was particularly focused on the data from 2,942 women of reproductive age, aged between 15 and 49 years.

Study Variables

The study examined the cooking fuel preferences of women of reproductive age, using these preferences as the dependent variable. Cooking fuel choices were categorized into two groups: the use of LPG as a cooking fuel (coded as +1) and the use of non-LPG options such as wood, straw, shrubs, grass, and charcoal (coded as -1). The study also considered several independent variables: type of residence (rural or urban), educational attainment (with or without formal education), and the wealth status (poor or non-poor) of the households where these women were surveyed.

Statistical Analysis

The Curie-Weiss Hamiltonian

Let N denote the population size of women of reproductive age, and we represent each individual's decision with a binary action:

$$\eta_a = \begin{cases} +1, & \text{if individual } a \text{ uses LPG as a cooking fuel,} \\ -1, & \text{if individual } a \text{ uses non-LPG as a cooking fuel .} \end{cases}$$

for a $\in \{1, \cdots, N\}$. We now define H_N for any $\eta \in \Omega_N = \{-1, +1\}^N$ as

$$H_N(\eta) = \frac{1}{2N} \sum_{a,b=1}^{N} \bar{J_{ab}} \eta_a \eta_b + \sum_{a=1}^{N} h_a \eta_a. \tag{0.1}$$

The notation $\eta = (\eta_1, \eta_2, \ldots, \eta_N) \in \Omega_N$ can be used to depict the choices made by all N individuals. $H_N(\eta)$ returns the level of satisfaction or utility of the entire population resulting from their choices represented by η. Higher values of $H_N(\eta)$ indicate greater satisfaction or utility within the population. The Hamiltonian $H_N(\eta)$ consists of two components: the first part models the social incentives of individuals in the population, while the second part models the private incentives of each individual [42]. In this context, η_a represents the choice made by an individual a, $\bar{J_{ab}}$ measures the influence that the individual a has on individual b which represents the strength of social incentive, and h_a represents the external influence. A positive value of $\bar{J_{ab}}$ indicates that conformity or imitation is rewarded, whereas a negative value suggests that imitation or conformity is not rewarded [26].

Multipopulation Curie-Weiss Model

Our main premise is that women of reproductive age with similar characteristics exhibit similar behaviour, whereas women of reproductive age with diverse attributes display diverse behaviours. This premise plays a crucial role in redefining the parameters of the Curie-Weiss Hamiltonian as shown in equation (0.1). Consequently, our primary objective is to determine a suitable parameterization for the interaction coefficient $\bar{J_{ab}}$ and develop a systematic approach for estimating the model's parameters using data. In line with our discrete choice model, each individual a is assigned a set of k characteristics:

$$T_a = \left(T_a^{(1)}, T_a^{(2)}, \ldots . T_a^{(k)} \right) \tag{0.2}$$

where the $T_a^{(j)} \in \{0, 1\}^k$ for $j = 1, 2, \cdots, k$. We consider the scenario in which the socio-economic attributes under examination are educational attainment represented by $T_a^{(1)}$, wealth status represented by $T_a^{(2)}$, and place of residence represented by $T_a^{(3)}$ with:

$$T_a^{(1)} = \begin{cases} 1, & \text{if woman } a \text{ has some form of education} \\ 0, & \text{if woman } a \text{ has no education} \end{cases} \tag{0.3}$$

$$T_a^{(2)} = \begin{cases} 1, & \text{if woman } a \text{ is non poor} \\ 0, & \text{if woman } a \text{ is poor} \end{cases} \tag{0.4}$$

and

$$T_a^{(3)} = \begin{cases} 1, & \text{if woman } a \text{ dwells in rural} \\ 0, & \text{if woman } a \text{ dwells in urban.} \end{cases} \tag{0.5}$$

If the value of an attribute is zero for an individual, then it means that a particular attribute does not contribute to the private incentive of that individual. Because h_a is

what motivates an individual to make a choice or decision, it is reasonable for it to become dependent on the vector of socio-economic attributes T_a since every rational person considers her status before making a choice or decision. Therefore, it can be written as a function of T_a as follows: let β_j for $j = 0, \cdots, k$ be the component of the vector $\beta = (\beta_0, \beta_1, \cdots, \beta_k)$ and assume β_j does not depend on the specific individual a. The vector β tells us the relative weight or importance that the various socio-economic attributes has or measures the private incentive for each attribute when an individual is making a decision. In our case this leads to

$$h_a = \sum_{j=1}^{k} \beta_j T_a^{(j)} + \beta_0. \tag{0.6}$$

Here, β_0 represents the fundamental private incentive that everyone person possesses, irrespective of their unique socio-economic characteristics [26].

Estimation

The least squares method is used to estimate the model parameters. As a result, we must identify the parameter settings that minimize

$$\sum_{g} [\bar{m}_g - \tanh(U_g)]^2 \tag{0.7}$$

where $\bar{m_g}$ is the average choice of group g. Because $\tanh(U_g)$ is non-linear, the computation will take an extremely long time , see [26]. In the interaction scenario, the independent variables are correlated. As a result, the least squares method is rendered ineffective. In that case, the partial least squares estimation method will be utilized. In the following section, we utilized the previously described estimation method to assess the parameters concerning the fuel choices of Ghanaian women of reproductive age. We would then estimate these parameters based on their socio-economic characteristics, including educational attainment, wealth status, and place of residence. Furthermore, the independent variables are correlated in the interaction scenario, rendering the least squares method ineffective. To address this issue, the partial least squares estimate would be employed [26]. We utilized MATLAB software version R2016a for carrying out our analyses and obtaining results.

In this research, we employed a statistical mechanical framework to investigate the relationships among socio-economic variables such as education level, wealth status, and living environment, and their impact on the choice of cooking fuels. We analyzed data from the 2019 Ghana Malaria Indicator Survey, which included responses from 2,942 women of reproductive age. The findings revealed that 2.92% of educated women of reproductive age from rural areas and 10.37% from urban areas, along with 2.58% of financially better-off women in rural settings and 10.54% in urban settings, chose LPG for cooking. In contrast, 37.15% of educated women in rural areas and 22.09% in urban areas, as well as 33.37% of wealthier women in rural settings and 10.47% in urban settings, preferred non-LPG fuels for their cooking needs.

Here we are looking at women of reproductive age that have been partitioned according to three attributes $T_a^{(1)}$, $T_a^{(2)}$ and $T_a^{(3)}$ representing educational attainment, wealth status, and place of residence. Table 1 shows the partition of the population into eight subsection, that is, women of reproductive age with some form of education and in rural, women of reproductive age with no education and in rural, women of reproductive age with some form of education and in urban, women of reproductive age with no education and in urban, women of reproductive age of reproductive age in rural and

non-poor, women of reproductive age in rural and poor, women of reproductive age in urban and non-poor and women of reproductive age in urban and poor.

Tables 2 and 3 below show the partitioning of the population on the use of LPG and non-LPG with respect to their attributes.

Table 1. Population classification based on attributes.

Attributes	Educational Attainment		Wealth Status	
Residence	Some form of education	No education	Non-poor	Poor
Rural	1179	615	175	1619
Urban	955	193	618	530

We got eight groups that were indexed with this type of partitioning $a = 1, 2, \cdots, 8$.
In particular,

$a = 1$ represents the group of women of reproductive age with some form of education and in rural,
$a = 2$ represents the group women of reproductive age with no education and in rural,
$a = 3$ represents the group of women of reproductive age with some form of education and in urban,
$a = 4$ represents the group of women of reproductive age with no education and in urban,
$a = 5$ represents the group of women of reproductive age in rural and non-poor,
$a = 6$ represents the group of women of reproductive age in rural and poor,
$a = 7$ represents the group of women of reproductive age in urban and non-poor,
$a = 8$ represents the group of women of reproductive age in urban and poor.

Table 2. LPG Users.

Attributes	Educational Attainment		Wealth Status	
Residence	Some form of education	No education	Non-poor	Poor
Rural	86	2	76	12
Urban	305	12	310	7

Table 3. Non-LPG (wood, straw/shrubs/grass , and charcoal) Users.

Attributes	Educational Attainment		Wealth Status	
Residence	Some form of education	No education	Non-poor	Poor
Rural	1093	613	99	1607
Urban	650	181	308	523

We then computed the values of the attributes $T_a^{(1)}$, $T_a^{(2)}$ and $T_a^{(3)}$ representing educational level, wealth status, and place of residence depending on the group a woman of reproductive age belongs, for the eight cases generated in Table 4 below. The values assigned to the attributes in each of the eight cases describe the relevance of that attribute to the private incentive part of the Hamiltonian. For instance in Case 1, being a woman of reproductive age with no form of education as an attribute does not contribute to the private incentive of a group, while being a woman of reproductive age with some form of education contributes to the private incentive of a group.

Table 4. Attributes of the population.

ATTRIBUTES						
cases	Educational Attainment($T_a^{(1)}$)		Wealth Status($T_a^{(2)}$)		Residence($T_a^{(3)}$)	
	No education	Some form of education	Poor	Non-Poor	Urban	Rural
1	0	1	1	0	1	0
2	1	0	0	1	1	0
3	0	1	0	1	0	1
4	1	0	1	0	0	1
5	0	1	1	0	1	0
6	1	0	0	1	1	0
7	0	1	0	1	0	1
8	1	0	1	0	0	1

Table 5. Estimates for the non-interacting model.

Cases	Estimate
Case 1: women of reproductive age with some form of education and in rural	$\beta_1 = 0.4012$<br>$\beta_2 = 0.1324$<br>$\beta_3 = -0.0523$<br>$\beta_0 = -0.4891$
Case 2: women of reproductive age with no education and in rural	$\beta_1 = -0.0815$<br>$\beta_2 = 0.1394$<br>$\beta_3 = -0.1280$<br>$\beta_0 = -0.1568$
Case 3: women of reproductive age with some form of education and in urban	$\beta_1 = 0.0575$<br>$\beta_2 = 0.1310$<br>$\beta_3 = 0.2914$<br>$\beta_0 = -0.4317$
Case 4: women of reproductive age with no education and in urban	$\beta_1 = -0.3371$<br>$\beta_2 = 0.0985$<br>$\beta_3 = -0.1103$<br>$\beta_0 = -0.0457$
Case 5: women of reproductive age in rural and non-poor	$\beta_1 = 0.4012$<br>$\beta_2 = 0.1324$<br>$\beta_3 = -0.0523$<br>$\beta_0 = -0.4891$
Case 6: women of reproductive age of reproductive age in rural and poor	$\beta_1 = -0.0815$<br>$\beta_2 = 0.1394$<br>$\beta_3 = -0.1280$<br>$\beta_0 = -0.1568$
Case 7: women of reproductive age of reproductive age in urban and non-poor	$\beta_1 = 0.0575$<br>$\beta_2 = 0.1310$<br>$\beta_3 = 0.2914$<br>$\beta_0 = -0.4317$
Case 8: women of reproductive age in urban and poor	$\beta_1 = -0.3371$<br>$\beta_2 = 0.0985$<br>$\beta_3 = -0.1103$<br>$\beta_0 = -0.0457$

Table 6. Estimates for the interacting model.

Parameter	Estimate
J_{11}	0.1203
J_{12}	0.0730
J_{13}	0.0412
J_{14}	0.0202
J_{15}	0.0027
J_{16}	0.1905
J_{17}	-0.0002
J_{18}	0.0616
J_{21}	0.0996
J_{22}	0.0604
J_{23}	0.0341
J_{24}	0.0167
J_{25}	0.0023
J_{26}	0.1578
J_{27}	-0.0002
J_{28}	0.0510

Parameter	Estimate
J_{31}	-0.1184
J_{32}	-0.0718
J_{33}	-0.0406
J_{34}	-0.0199
J_{35}	-0.0027
J_{36}	-0.1875
J_{37}	0.0002
J_{38}	-0.0607
J_{41}	-0.0620
J_{42}	-0.0376
J_{43}	-0.0213
J_{44}	-0.0104
J_{45}	-0.0014
J_{46}	-0.0982
J_{47}	0.0001
J_{48}	-0.0318

Parameter	Estimate
J_{51}	-0.0072
J_{52}	-0.0044
J_{53}	-0.0025
J_{54}	-0.0012
J_{55}	-0.0002
J_{56}	-0.0114
J_{57}	0.0000
J_{58}	-0.0037
J_{61}	0.2118
J_{62}	0.1285
J_{63}	0.0726
J_{64}	0.0355
J_{65}	0.0048
J_{66}	0.3354
J_{67}	-0.0004
J_{68}	0.1085

Parameter	Estimate
J_{71}	-0.1329
J_{72}	-0.0806
J_{73}	-0.0455
J_{74}	-0.0223
J_{75}	-0.0030
J_{76}	-0.2105
J_{77}	0.0003
J_{78}	-0.0681
J_{81}	-0.1111
J_{82}	-0.0674
J_{83}	-0.0381
J_{84}	-0.0187
J_{85}	-0.0025
J_{86}	-0.1760
J_{87}	0.1002
J_{88}	-0.1570
β_1	1.311
β_2	0.9678
β_3	0.7887
β_0	0

Results

Non interacting Case

The group's decision to use LPG or non-LPG as cooking fuel depends on whether the sum of the individual private incentives, β_j, is positive or negative. A positive sum will lead to the choice of LPG, while a negative sum will result in the selection of non-LPG. The calculated estimates for β_j are shown in Table 5. The sums of β_j for cases 1, 2, 4, 5, 6, and 8 are negative, indicating that individuals in these cases are more likely to choose non-LPG as their cooking fuel when there is no social interaction. Conversely, the positive sums of β_j in cases 3 and 7 suggest that women of reproductive age are inclined

to opt for LPG as their cooking fuel in the absence of social interaction.

If social interaction is absent, the estimates suggest that wealth status (β_2) is related to the choice of cooking fuel, as the private incentive for wealth status is positive in Cases 1 through 8. The negative value of the base private incentive (β_0) in all eight instances indicates that individuals would opt for some form of non-LPG cooking fuel option if there is no social interaction present.

Interacting Case

In the interactive model, the utility function considers both social and private incentives. If the social incentive, $\bar{J_{ab}}$, is positive, individuals within the groups tend to mimic each other's choices. Conversely, when $\bar{J_{ab}}$ takes negative values, women of reproductive age in those groups are likely to make dissimilar choices instead of adhering to the norm. The decision to use LPG or non-LPG as cooking fuel depends on the sum of the private incentives, represented by β_j. If this sum is positive, the group will opt for LPG; if negative, they will choose non-LPG.

Note that $\bar{J_{11}}$ represents the strength of interaction among women of reproductive age, with some form of education and living in rural areas, interacting with each other. As shown in Table 6, the estimated value of $\bar{J_{11}}$ is positive, indicating that imitation is considered a reward in this group. On the other hand, $\bar{J_{33}}$ refers to the strength of interaction among women of reproductive age with some form of education, living in urban areas, interacting with each other. The estimated value of $\bar{J_{33}}$ is negative, suggesting that imitation is not seen as a reward in this group. $\bar{J_{61}}$ has the highest estimate for social interaction, indicating that women of reproductive age in rural areas, who are poor, have a greater influence on their educated peers also living in rural areas.

Table 5 shows that educational attainment, wealth status, and place of residence have a relationship with the choice of cooking fuel when social interaction is present, as indicated by the positive values of β_1, β_2, and β_3. Additionally, the value of β_0 is zero, which implies that in situations where there is social interaction, women of reproductive age consider their education, wealth status, or residence when deciding whether to use LPG as a cooking fuel.

Discussions

In this study, we explored the relationship between socio-economic factors and cooking fuel choices in Ghana, using data from the 2019 Ghana Malaria Indicator Survey (GMIS). Our findings revealed a relationship between educational attainment, wealth status, place of residence, and the adoption of liquefied petroleum gas (LPG) as a cleaner alternative for cooking fuel when social interactions are considered.

In line with existing literature, our study highlights a positive correlation between education and the use of LPG for cooking. Women of reproductive age in urban areas with some form of education show a significant preference for LPG, emphasizing the role of education in promoting environmental awareness and the health benefits of cleaner cooking fuels [45]. This aligns with our observation that 2.92% of women of reproductive age in rural areas and 10.37% of women in urban areas with some form of education opted for LPG, compared to the higher usage rates of non-LPG fuels among their less educated counterparts. Moreover, our analysis indicates that wealth status is related to cooking fuel choices, with wealthier households more likely to adopt LPG. This finding corroborates the research by [46] and [47], who noted economic barriers as a critical factor hindering the transition to cleaner cooking fuels in developing countries.

The role of social interactions in fuel choice decisions presents a complex picture. Our study suggested that in rural settings, poorly educated women of reproductive age significantly influence their uneducated peers regarding cooking fuel choices. This dynamic warrants further investigation to inform targeted interventions.

Addressing the study's limitations, we acknowledge that our reliance on cross-sectional data from the Ghana Multiple Indicator Survey (GMIS) limits our ability to infer causal relationships. Future research could benefit from longitudinal studies that explore how changes in socio-economic status over time affect cooking fuel choices. Given the clear relationship between socio-economic factors, such as educational attainment, wealth status, place of residence and cooking fuel choices, policies aimed at promoting cleaner cooking fuels in Ghana must consider economic incentives, provide free education for women of reproductive age, and implement community-based interventions tailored to the specific needs of rural and urban populations.

In conclusion, our study contributes to the growing body of evidence that supports the need for multifaceted approaches to encourage the adoption of cleaner cooking fuels in Ghana. Further research should explore the impact of social networks and community-led programs on promoting environmental sustainability and public health through cleaner cooking practices.

Model Diagnostics and Validation

In this section, we will assess the goodness of fit of our interacting model to the data. To estimate the parameters of the interacting model proposed in [43], we employed the partial least squares (PLS) method. Our dependent variable is represented by U_g, while our independent variable is $\bar{m}_g$. PLS is a technique used to predict a set of dependent variables based on a set of independent variables, which results in orthogonal factors or latent vectors derived from the independent variables. These latent vectors are then sorted by decreasing order of their eigenvalues, and the number of latent vectors used is determined by those that best explain the covariance between the independent and dependent variables [44].

Table 7 displays the variance explained by the latent vectors used in estimating the independent variables ($\bar{m}_g$) and the dependent variable (U_g), as well as their root mean square error of prediction (RMSEP). According to Table 7, the first seven latent vectors explain 97.31% of the variance in U_g and 94.70% of the variance in $\bar{m}_g$. The variances explained by both the dependent and independent variables are sufficient for modelling or prediction. The RMSEP decreases as the number of latent vectors increases until it reaches the sixth latent vector, at this point it increases again for both $\bar{m}_g$ and U_g.

Table 7. Variance of $\bar{m}_g$ and U_g explained by the latent vectors and RMSEP

Latent Vectors	Percentage of Explained Variances for $\bar{m}_g$	Cumulative Percentage of Explained Variances for $\bar{m}_g$	Percentage of Explained Variances for U_g	Cumulative Percentage of Explained Variances for U_g	RMSEP for $\bar{m}_g$	RMSEP for U_g
1	27.47	27.47	39.84	39.84	1.0761	0.1918
2	10.89	38.36	15.98	55.82	0.8509	0.1054
3	9.84	48.2	7.84	63.66	0.7349	0.0548
4	10.54	58.74	0.34	64.00	0.6528	0.01
5	5.42	64.16	0	64.00	0.4339	0
6	14.78	78.94	16.11	80.11	0.3543	0
7	15.76	94.70	17.20	97.31	0.5282	0.07681

Conclusion

This study provided critical insights into the adoption of liquefied petroleum gas (LPG) compared to non-LPG fuels among various socio-economic segments in Ghana. We identified a significant statistical relationship between socio-economic factors—specifically, educational attainment, wealth status, and place of residence—and choices of cooking fuel. This relationship is shaped by both individual socio-economic attributes and broader social interactions.

Our findings suggest that although education and wealth positively correlate with the use of cleaner cooking fuels like LPG, social dynamics play a crucial role in fuel selection. Notably, in urban areas, our study did not show a significant imitative effect among peers, contrary to expectations set by previous research. However, in rural areas, educated women significantly influence their less-educated peers towards adopting cleaner fuels, highlighting the critical role of targeted educational interventions.

Furthermore, our study indicated a stronger propensity for the adoption of LPG among women in urban areas, regardless of their economic status, compared to their rural counterparts. This suggests that infrastructure and accessibility issues are likely less constraining in urban settings. Interestingly, urban poor women appear to exert considerable influence over their non-poor peers, suggesting a potential for harnessing this influence in broader awareness and adoption programs.

Given these insights, we recommend that policy interventions in Ghana focus on leveraging the influential capacity of both urban poor women and educated rural women. Initiatives such as community-led programs, enhanced educational campaigns, and financial support mechanisms, including microfinance and subsidies, could be pivotal. Additionally, establishing reliable LPG supply infrastructure and tailoring communication strategies to foster peer-to-peer learning and collaboration with local institutions could accelerate the shift towards cleaner cooking fuels. By integrating these strategies, we aim to enhance public health, protect the environment, and contribute to economic growth through the increased adoption of clean and efficient cooking fuels.

Funding

The author(s) received no specific funding for this work.

Availability of data and materials

The dataset used in this study is included in the supplementary file.

Ethics approval and consent to participate

The ethical protocols for the 2019 Ghana Maternal Health Survey (GMIS) received approval from the Ghana Health Service Ethical Review Committee and the ICF International Institutional Review Board. Participation in this survey was entirely voluntary, with participants being fully informed about the potential risks and benefits involved. Only those who consented to participate by signing an informed consent form were included in the study. There was no specific ethics ID or approval number assigned. Participants were required to give their informed consent before they could take part in the study.

Competing interests

The author declares that he has no competing interests.

References

1. Choudhuri P, Desai S. Gender inequalities and household fuel choice in India. Journal of cleaner production. 2020 Aug 20;265:121487.
2. Foell W, Pachauri S, Spreng D, Zerriffi H. Household cooking fuels and technologies in developing economies. Energy policy. 2011 Dec 1;39(12):7487-96.
3. AFRICA SS. Energy Outlook.
4. World Health Organization. World health statistics 2017: monitoring health for the SDGs. sustainable development goals. 2017 Nov;7.
5. Ghana. Statistical Service. Ghana living standards survey. Ghana Statistical Service; 1991.
6. Muller C, Yan H. Household fuel use in developing countries: Review of theory and evidence. Energy Economics. 2018 Feb 1;70:429-39.
7. Anang BT, Akuriba MA, Alerigesane A. Charcoal production in Gushegu District, Northern Region, Ghana: lessons for sustainable forest management. International Journal of Environmental Sciences. 2011;1(7):1944-53.
8. Prasad G. Improving access to energy in sub-Saharan Africa. Current Opinion in Environmental Sustainability. 2011 Sep 1;3(4):248-53.
9. Bond TC, Sun H. Can reducing black carbon emissions counteract global warming?.
10. Austin KF, Mejia MT. Household air pollution as a silent killer: women's status and solid fuel use in developing nations. Population and Environment. 2017 Sep;39:1-25.

11. Pope DP, Mishra V, Thompson L, Siddiqui AR, Rehfuess EA, Weber M, Bruce NG. Risk of low birth weight and stillbirth associated with indoor air pollution from solid fuel use in developing countries. Epidemiologic reviews. 2010 Apr 1;32(1):70-81.

12. Amegah AK, Quansah R, Jaakkola JJ. Household air pollution from solid fuel use and risk of adverse pregnancy outcomes: a systematic review and meta-analysis of the empirical evidence. PloS one. 2014 Dec 2;9(12):e113920.

13. Epstein MB, Bates MN, Arora NK, Balakrishnan K, Jack DW, Smith KR. Household fuels, low birth weight, and neonatal death in India: the separate impacts of biomass, kerosene, and coal. International journal of hygiene and environmental health. 2013 Aug 1;216(5):523-32.

14. Corsini E, Vecchi R, Marabini L, Fermo P, Becagli S, Bernardoni V, Caruso D, Corbella L, Dell'Acqua M, Galli CL, Lonati G. The chemical composition of ultrafine particles and associated biological effects at an alpine town impacted by wood burning. Science of the total environment. 2017 Jun 1;587:223-31.

15. Asante KP, Afari-Asiedu S, Abdulai MA, Dalaba MA, Carrión D, Dickinson KL, Abeka AN, Sarpong K, Jack DW. Ghana's rural liquefied petroleum gas program scale up: A case study. Energy for Sustainable Development. 2018 Oct 1;46:94-102.

16. Ahunu LI. LPG promotion program. Africa Centre for Energy Policy. 2015 Oct.

17. Karimu A, Mensah JT, Adu G. Who adopts LPG as the main cooking fuel and why? Empirical evidence on Ghana based on national survey. World Development. 2016 Sep 1;85:43-57.

18. Bofah RO, Appiah-Konadu P, Ngwu FN. Transition to cleaner cooking energy in Ghana. Clean Energy. 2022 Feb 1;6(1):193-201.

19. Gould CF, Urpelainen J, Sais JH. The role of education and attitudes in cooking fuel choice: Evidence from two states in India. Energy for Sustainable Development. 2020 Feb 1;54:36-50.

20. Gould CF, Urpelainen J. The gendered nature of liquefied petroleum gas stove adoption and use in rural India. The Journal of Development Studies. 2020 Jul 2;56(7):1309-29.

21. Durlauf SN. How can statistical mechanics contribute to social science?. Proceedings of the national academy of sciences. 1999 Sep 14;96(19):10582-4.

22. Brock W, Durlauf SN. Discrete choice with social interactions I: Theory.

23. Brock WA, Durlauf SN. Discrete choice with social interactions. The Review of Economic Studies. 2001 Apr 1;68(2):235-60.

24. Ermentrout B, Terman DH. Mathematical foundations of neuroscience. New York: springer; 2010 Jul 1.

25. Turchin P, Taylor AD. Complex dynamics in ecological time series. Ecology. 1992 Feb;73(1):289-305.

26. Opoku AA, Osabutey G, Kwofie C. Parameter evaluation for a statistical mechanical model for binary choice with social interaction. Journal of Probability and Statistics. 2019 Mar 4;2019.

27. Kuran T. Now out of never: The element of surprise in the East European revolution of 1989. World politics. 1991 Oct;44(1):7-48.

28. Fishwick MW. Critical mass: how one thing leads to another. The Journal of American Culture. 2004 Dec 1;27(4):438.

29. Pra PD, Giacomin G, Regoli D. Noise-induced periodicity: some stochastic models for complex biological systems. Mathematical Models and Methods for Planet Earth. 2014:25-35.

30. Collet F, Dai Pra P, Formentin M. Collective periodicity in mean-field models of cooperative behavior. Nonlinear Differential Equations and Applications NoDEA. 2015 Oct;22:1461-82.

31. Collet F, Dai Pra P, Sartori E. A simple mean field model for social interactions: dynamics, fluctuations, criticality. Journal of Statistical Physics. 2010 Jun;139:820-58.

32. Frey R, Backhaus J. Pricing and hedging of portfolio credit derivatives with interacting default intensities. International Journal of Theoretical and Applied Finance. 2008 Sep;11(06):611-34.

33. Di Biasio A, Agliari E, Barra A, Burioni R. Mean-field cooperativity in chemical kinetics. Theoretical Chemistry Accounts. 2012 Mar;131:1-4.

34. Volkov I, Banavar JR, Hubbell SP, Maritan A. Neutral theory and relative species abundance in ecology. Nature. 2003 Aug 28;424(6952):1035-7.

35. Schiffer P, Daruka I. Two-population model for anomalous low-temperature magnetism in geometrically frustrated magnets. Physical Review B. 1997 Dec 1;56(21):13712.

36. Gallo I, Contucci P. Bipartite mean field spin systems. Existence and solution. arXiv preprint arXiv:0710.0800. 2007 Oct 3.

37. Gallo I, Barra A, Contucci P. Parameter evaluation of a simple mean-field model of social interaction. Mathematical Models and Methods in Applied Sciences. 2009 Aug;19(supp01):1427-39.

38. Osabutey G, Opoku AA, Gyamfi S. A statistical mechanics approach to the study of energy use behaviour. Journal of applied mathematics. 2020 May 1;2020:1-4.

39. Amegah AK, Jaakkola JJ, Quansah R, Norgbe GK, Dzodzomenyo M. Cooking fuel choices and garbage burning practices as determinants of birth weight: a cross-sectional study in Accra, Ghana. Environmental Health. 2012 Dec;11:1-0.

40. Weber E, Adu-Bonsaffoh K, Vermeulen R, Klipstein-Grobusch K, Grobbee DE, Browne JL, Downward GS. Household fuel use and adverse pregnancy outcomes in a Ghanaian cohort study. Reproductive health. 2020 Feb 22;17(1):29.

41. Measure DH. The DHS program: demographic and health surveys. Rockville: ICF Macro. 2016.

42. Thompson CJ. Mathematical statistical mechanics. Princeton University Press; 2015 Mar 8.

43. Contucci P, Gallo IA. Bipartite mean field spin systems. Existence and solution. MPEJ. 2008;14:1-22.

44. Abdi H. Partial least squares regression and projection on latent structure regression (PLS Regression). Wiley interdisciplinary reviews: computational statistics. 2010 Jan;2(1):97-106.

45. Timilsina RR, Rahut DB, Gautam MS, Mishra R, Sonobe T. Are households shifting toward cleaner cooking fuel? Empirical evidence from India during 2005-2021. Frontiers in Environmental Economics. 2023;2:1137248.

46. Vigolo V, Sallaku R, Testa F. Drivers and barriers to clean cooking: a systematic literature review from a consumer behavior perspective. Sustainability. 2018 Nov 21;10(11):4322.

47. Puzzolo E, Pope D, Stanistreet D, Rehfuess EA, Bruce NG. Clean fuels for resource-poor settings: A systematic review of barriers and enablers to adoption and sustained use. Environmental research. 2016 Apr 1;146:218-34.

Supporting information

S1 Dataset. **Supporting Information on the Dataset**.

S2 Methodology. **Supplementary Information on the Methodology**.

Supplementary materials

Click here to access/download
Supporting Information
Supplementary Materials.pdf
